# Analysing mHealth usage logs in RCTs: Explaining participants’ interactions with type 2 diabetes self-management tools

**DOI:** 10.1371/journal.pone.0203202

**Published:** 2018-08-30

**Authors:** Meghan Bradway, Gerit Pfuhl, Ragnar Joakimsen, Lis Ribu, Astrid Grøttland, Eirik Årsand

**Affiliations:** 1 Norwegian Centre for E-health Research, University Hospital of North Norway, Tromsø, Norway; 2 Department of Clinical Medicine, UiT The Arctic University of Norway, Tromsø, Norway; 3 Department of Psychology, UiT The Arctic University of Norway, Tromsø, Norway; 4 Department of Internal Medicine, University Hospital of North Norway, Tromsø, Norway; 5 Department of Nursing and Health Promotion, Oslo Metropolitan University, Oslo, Norway; Universiteit Twente, NETHERLANDS

## Abstract

**Background:**

The Introduction of mobile health (mHealth) devices to health intervention studies challenges us as researchers to adapt how we analyse the impact of these technologies. For interventions involving chronic illness self-management, we must consider changes in behaviour in addition to changes in health. Fortunately, these mHealth technologies can record participants’ interactions via usage-logs during research interventions.

**Objective:**

The objective of this paper is to demonstrate the potential of analysing mHealth usage-logs by presenting an in-depth analysis as a preliminary study for using behavioural theories to contextualize the user-recorded results of mHealth intervention studies. We use the logs collected by persons with type 2 diabetes during a randomized controlled trial (RCT) as a use-case.

**Methods:**

The Few Touch Application was tested in a year-long intervention, which allowed participants to register and review their blood glucose, diet and physical activity, goals, and access general disease information. Usage-logs, i.e. logged interactions with the mHealth devices, were collected from participants (n = 101) in the intervention groups. HbA1c was collected (baseline, 4- and 12-months). Usage logs were categorized into registrations or navigations.

**Results:**

There were n = 29 non-mHealth users, n = 11 short-term users and n = 61 long-term users. Non-mHealth users increased (+0.33%) while Long-term users reduced their HbA1c (-0.86%), which was significantly different (*P* = .021). Long-term users significantly decreased their usage over the year (*P* < .001). K-means clustering revealed two clusters: one dominated by diet/exercise interactions (n = 16), and one dominated by BG interactions and navigations in general (n = 40). The only significant difference between these two clusters was that the first cluster spent more time on the goals functionalities than the second (*P* < .001).

**Conclusion:**

By comparing participants based upon their usage-logs, we were able to discern differences in HbA1c as well as usage patterns. This approach demonstrates the potential of analysing usage-logs to better understand how participants engage during mHealth intervention studies.

## Introduction

Standard approaches for evaluating research activities do not sufficiently address all aspects of mobile health (mHealth) interventions. This is in part due to a reliance on hard clinical endpoints, and part because study designs often follow the “Black Box evaluation” method [1], the aim of which is to answer «what» has changed, in retrospect, by comparing end- and baseline measures. In doing so, these studies traditionally produce evidence of, for example, how a new medication will predictably affect individuals with a certain diagnosis in real-world medical practice. For pharmacology, this is acceptable. The usefulness of these results is clear; either the drug is safe and efficient to use, or not. However, intervention studies for chronic illnesses that utilized modern technologies often conclude that their results require further testing [2–4]. This is, in part, due to the complicated nature of chronic illness self-management, requiring individuals’ to make daily health decisions in response to biological changes, such as their blood glucose in the case of diabetes. Humans, their decisions and behaviours require greater understanding than biology alone. Therefore, clinical research must adapt to look at how participants choose to behave during the intervention in order to understand why an intervention is–or is not–producing any effects. In other words, tracking and understanding participants’ behavior can allow us to understand what is going on inside “The Black Box” during intervention studies.

Today, many who live with diabetes rely on such mHealth devices as smartphones and wearable trackers to aid them in their self-management. These tools can reduce the burden of performing self-management by allowing individuals to more easily record and review their self-management measures, i.e. blood glucose, physical activity, diet and medication at their fingertips. Fortunately for clinical research, these technologies can also provide date-stamped records of a user’s self-management decisions through their interactions with the mHealth devices, i.e. usage-logs, and registered health measures, lifestyle habits, and notes [5, 6]. However, due to the novelty of these technologies for health care, there is no standard for how to assess these newly available data. Recent studies have, to a certain degree, incorporated analysis of usage log patterns during clinical trials. Some have even included values of registered health data [7]. However, most of these have only analysed cumulative measures such as total keystrokes or hours interacting with a device [8, 9], and many are inconclusive and fail to contextualize the data.

Therefore, mHealth research calls for a toolbox of sorts–in addition to traditional measures, a collection of concepts adapted to inform new possibilities for explaining the impact of mHealth interventions. Approaches such as theory-driven evaluation [1], program theory evaluation using logic models [10], and logic analysis [11] have been proposed as alternatives to “Black Box evaluation”. Traditionally, these approaches are used to evaluate e.g. an educational program intervention. These programs consist of complex interactions between inputs and outputs. Theory-driven evaluation is used to explain either part or the whole of these contexts [12]. These approaches can inform the assessment of mHealth interventions because they aim to explain the context of an intervention, such as participants’ behaviour and decisions, rather than to predict cause-and-effect, such as the effect of a drug.

For the case of mHealth interventions for diabetes, we must consider the context. Individuals are expected to continuously self-manage their diabetes through a cycle of trial and error. The cycle is characterized by tracking, reflecting upon, reacting and repeating certain health actions, which personifies the behavioural theories of Experiential Learning [13] and Health Habit Change [14–19]. Therefore, the registered data and usage logs that track these actions, available on the mHealth devices, can be interpreted as reflections of an individuals’ engagement in their health. This provides a much more detailed picture of how patients are relating to mHealth over the course of an intervention.

In this paper, we present a preliminary study for applying human behaviour theories [13, 18–20] to structure and analyse usage logs. We used the use-case of the logs collected by the mHealth intervention used in the REgioNs of Europe WorkINg toGether for HEALTH (RENEWING HEALTH) Norwegian randomized control trial (RCT).

## Objectives and aims

The overall aim is to provide evidence for how applying behavioural theories to usage logs can be used to provide a better understanding of the context of mHealth interventions. In doing so, we aim to inform the appropriate and effective design and administration of future mHealth studies.

## Methods

### Use case: The RENEWING HEALTH RCT

To demonstrate the potential benefits of analysing usage-logs to explain the impact of mHealth, we use the case of the European Commission funded RENEWING HEALTH project’s Norwegian RCT. The study was registered with Clinical Trials, with reference number NCT01315756, and was approved by the Regional Committee for Medical and Health Research Ethics in South-Eastern Norway (REK sør-øst).

**Fig 1:**
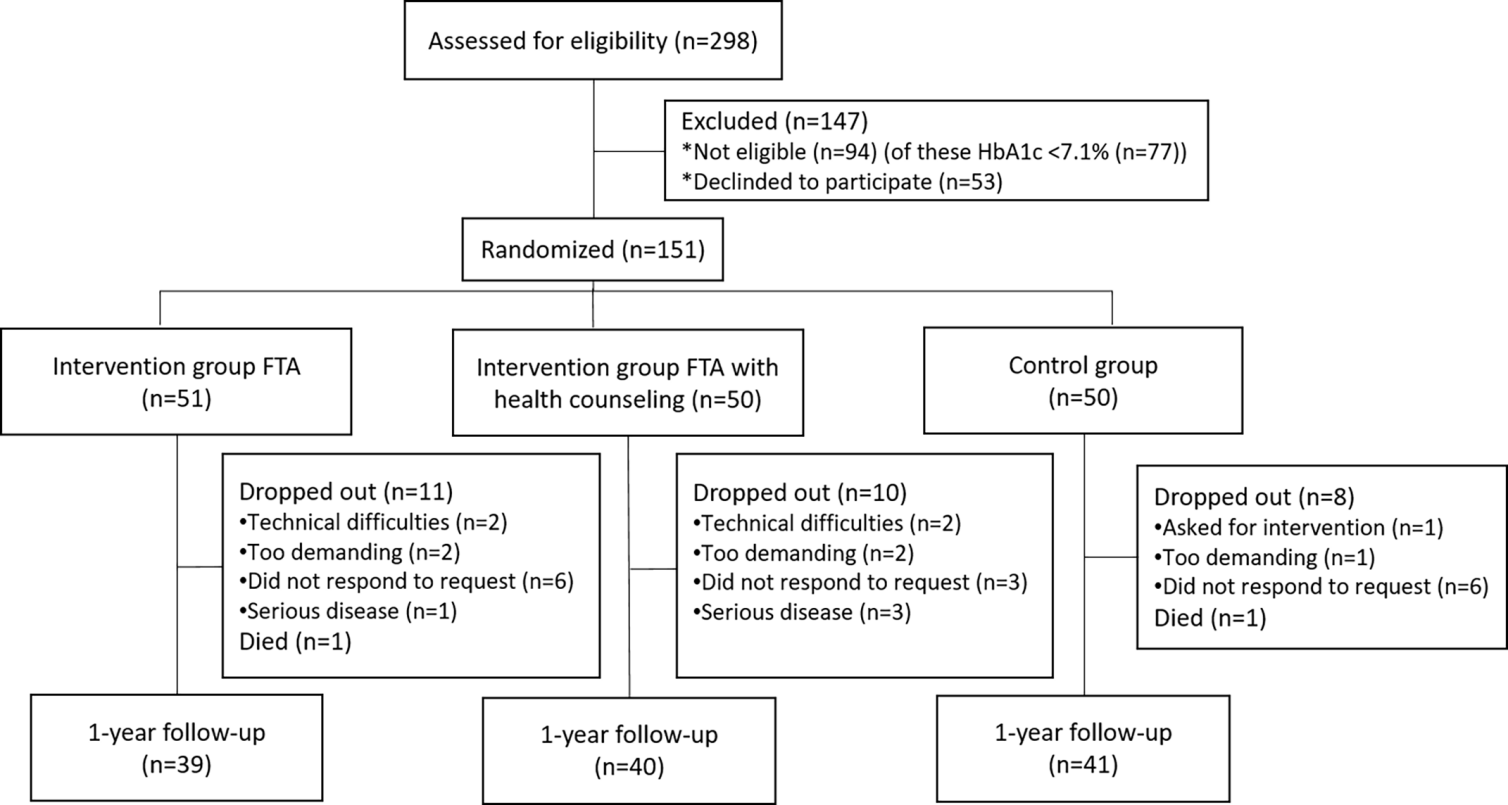
CONSORT flow diagram of the RCT.

This 3-armed study was conducted between 2011 and 2013 to test the impact of a mHealth self-management intervention called the Few Touch Application (FTA) [21], including use of a smartphone application (app) and glucose meter. The FTA intervention tracked when participants registered and reviewed their blood glucose, diet and physical activity, goals as well as accessed general disease information stored within the application. The app was Bluetooth-paired with the OneTouch Ultra Easy blood glucose meter from LifeScan through a Bluetooth adapter from Polymap Wireless, enabling fully automated transfer of BG measurements to the app. Originally, n = 151 participants were recruited and randomized into two intervention groups: n = 51 used the mHealth intervention (referred to as FTA); n = 50 used the mHealth intervention together with health counselling (referred to as FTA+HC); and a control group (n = 50) (Fig 1). The FTA+HC group was followed up by the diabetes nurse five times, remotely by phone, within the first 4 months. The diabetes nurse provided health counselling with principles from motivational interviewing and supported patients’ use of the FTA. Patients’ own registered app data and app usage were continuously gathered and stored. More detailed descriptions of the study design can be found in the protocol paper published elsewhere [22] (S1 and S2 Files).

#### Training

Both intervention groups were trained on how to use the mHealth intervention at the start of the study. Participants were provided both a paper and an electronic version (USB memory stick) of the user-guide. Included were explanations and screen shots of all app functionalities and step-by-step instructions for registering data using the provided mHealth system, both manually and using the system for automatic data transfer when using the blood glucose meter. The participants also had access to a technical support-service during work hours (9 AM-3 PM), by phone, in the study period.

#### Previous analysis and results

The primary analysis results for this study have been reported elsewhere [22–25]. These focused on comparing changes in HbA1c and questionnaire responses, with a coarse look at usage patterns. Of the 101 who received the mHealth intervention, with and without health counselling, 79 completed the study. It is important to note that the previous analysis was based upon the participants’ completion of the primary outcome (HbA1c level), and did not distinguish between their mHealth usage.

Previous results yielded only a significant increase in self-management related to “skill and technique acquisition” in the FTA with health counselling group compared to the control group. There was no significant difference in HbA1c or total usage of the mHealth devices at 4- and 12-months between those who received the mHealth intervention and the control group [23, 26]. Recently, a follow-up analysis for this study, found that half of the persons were in pre-action phase according to the stage of behaviour change, reflected in the physical activity and dietary-related usage patterns [25]. This demonstrates the potential of applying theories, related to the context of self-management, to the analysis of mHealth usage logs.

### An in-depth analysis of log-data

The mHealth usage logs from the RENEWING HEALTH study contained data and time stamps related to use of blood glucose- (BG), physical activity-, diet- functionalities and access to other disease information within the FTA smartphone app. In addition, the app automatically recorded when and what users registered as well as when they reviewed their past registrations or any other interaction with the app. By interacting with the app in these two ways, the user personifies the cycle of experiential learning by performing active engagement and self-reflection related to their self-management.

#### Population: Comparison of users vs. Non-users

As mentioned in the original protocol, we focus on those who participated in the intervention [22]. Therefore, in this descriptive analysis of the usage logs, we focus on those who used the mHealth devices. Because there were no statistical differences between the two FTA intervention groups, we consider all the 101 intervention participants as one cohort. We were then able to a) identify those who actually used the mHealth tools at any point in time during the study and b) explore and differentiate mHealth usage patterns in this presented in-depth analysis (S3 File).

Participants were included based upon whether or not, and for how long, they used the mHealth tools. To be considered as a user of the mHealth intervention, as opposed to one who casually explored the functionalities but chose not to continue using the tools, participants must have logged at least 60 interactions with the tool at any time in the 12 months, i.e. a minimum of 5 interactions per month. To be defined as Long-term users, participants must have used the FTA app for three or more continuous months, with at least 5 interactions per month. Because participants used the separate LifeScan BG meter with the adapter for automatic transfer of BG measurements, only functionalities that required user-interaction with the FTA smartphone app were included when categorizing the participants into the three groups: those who did not use the mHealth tools once (“Non-mHealth users”), those who used them for less than 3-continuous months (“Short-term users”) and those who used them for three or more continuous months (“Long-term users”).

Of the 101 participants who received the mHealth intervention, 29 participants fell in the “Non-Users” group, while 72 participants interacted with the mHealth devices at least once, and 61 of those participants used the devices for a minimum of three continuous months during the intervention period.

#### Measures: mHealth-usage logs

To describe how the theory of experiential learning was used to structure and interpret the usage logs, we grouped usage logs into two basic types: “Registrations”, which are an individual’s active interaction with their health through entry of self-management recordings into the app, and “Navigations”, which are any non-registration, or reflective, interactions with the app.

Due to an error in the logging routine, we were unable to distinguish “Goal registrations” from navigations. However, the number of minutes spent watching the app’s various screens were collected by the system. The logs were therefore grouped as follows:

- Diet/Exercise registrations (D/E Regs): indicating when a user manually registers information related to diet or physical activity.
- Diet/Exercise navigations (D/E Navs): when a user accesses previously registered data related to diet or physical activity, thereby demonstrating actions relevant for self-reflection.
- Blood glucose registrations (BG Regs): when a user measures blood glucose levels via the BG meter.
- Blood glucose navigations (BG Navs): when a user reviews previously measured blood glucose values, thereby demonstrating actions relevant for self-reflection upon past BG levels.
- Disease Informational navigations (Info Navs): when a user accesses disease information, thereby demonstrating actions relevant for active learning behaviour.

In addition, minutes spent per screen were calculated for the following screens: Home Screen, Data Navigations, and Goals (S1 Text).

#### Identifying emergent user subgroups

We used the FTA usage logs from the first three months, i.e. the first quarter, to identify usage patterns. Of the six main functionalities the FTA provided, a range of different usage patterns are possible (Table 1). We based the usage groups on the two most frequently used FTA functions. Conceptually, a patient may use the FTA mainly for registering self-management habits or also for reflections / navigating through previously registered health information. The FTA may also be used mainly for diet / exercise management, or blood glucose management. A range of other combinations is also possible. To investigate this in our sample, we employed k-means clustering (S2 Text).

**Table 1:** Differentiation of the 6 Usage groups based on two most used FTA functions.

	Diet/Exercise registrations	Diet/Exercise navigations	Blood Glucose registrations	Blood Glucose navigations	Goals registrations and navigations	Disease information navigations
**Registrations usage group**	X		X			
**Navigations usage group**		X		X		X
**Diet/Exercise management usage group**	X	X				
**Blood Glucose management usage group**			X	X		
**Goals usage group**					X	
**Inconsistent usage group**	Any combination of functionalities not otherwise described					

### Statistical analysis

In the first part of our analysis, we compared app-users to Non-app users. In the second part we focused the analysis on those who used the app for three or more consecutive months, i.e. Long-term users. The details of extracting and analysing of the log data can be found in the supplementary material (S1 Text).

To determine differences between participants based on overall duration of mHealth use, demographics, baseline HbA1c as well as interactions and logged time spent with the interventions’ mHealth tools’ usage were compared between Non-users (n = 29), Short-term users (n = 11) and Long-term users (n = 61) (Table 2).

**Table 2:** Descriptives for the three FTA usage groups.

*M (SD)*	Non mHealth users (N = 29)	Short-term users (N = 11)	Long-term users (N = 61)	F-value	*P*	η^2^
Gender	17 female	5 female	37 female			
Age	57.45 (12.97)	55.18 (12.86)	58.84 (11.26)	.49	.62	.01
Duration (years)	9.69 (7.87)	11.27 (7.14)	9.25 (8.3)	.30	.74	.01
Education (years)	3.72 (1.19)	3.91 (1.38)	3.61 (1.48)	.25	.78	.01
SMBG* (per week)	7.17 (7.315)	5.5 (5.11)	9.43 (10.46)	1.18	.31	.02
HbA1c at baseline	8.41 (1.11)	7.99 (.062)	8.08 (1.17)	1.01	.37	.02

*: SMBG is self-monitoring of blood glucose

**Fig 2:**
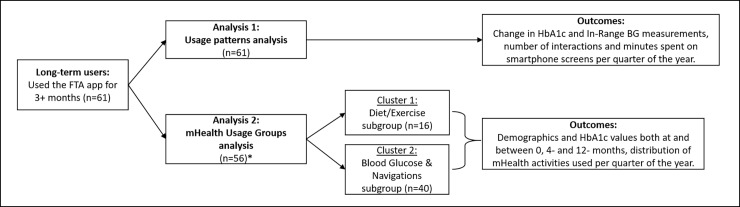
Diagram showing two approaches for analysing the usage logs. *Five participants were grouped in two very small clusters and not considered for Analysis 2.

The remaining tests of these in-depth analyses focused upon investigating relationships between patterns of app usage, and health outcomes from the Long-Term users (n = 61) (Fig 2). In addition to comparing usage patterns based on emergent groups, we also explored the smartphone app’s recorded blood glucose levels. Because the goal of blood glucose self-management (SMBG) is to keep BG levels within a certain range (4-10mmol/L), we chose to compare the number of In-Range BG values both within and between quarters.

Pearson Correlation and Repeated Measures ANOVA were used to compare change in HbA1c and app-usage activities (total as well as individual Diet/Exercise and BG Registrations and Navigations and Disease information Navigations) within and between groups, over the 12-months pooled into 3-month intervals (quarters of the year). Pearson Correlation analysis and Linear regression were used to analyse relationships between interactions with app-activities and minutes used on each app-screen over the four quarters. We have chosen the pooling into quarters as it reduced the number of missing values compared to a monthly or bi-monthly analysis. The statistical package for social sciences (SPSS) version 19 software, and JASP version 0.8.5 [27] were used to run the statistical tests.

### Ethics

This study was approved by the Regional Committee for Medical and Health Research Ethics in South-Eastern Norway (reference number 2010/3386). All patients provided signed informed consent documents before participation in the intervention. If patients revoked their consent, their data was removed from the database and not included in analysis.

## Results

As seen in Table 2, there was no selection bias between those who did and did not use the mHealth intervention tools for self-management.

**Fig 3:**
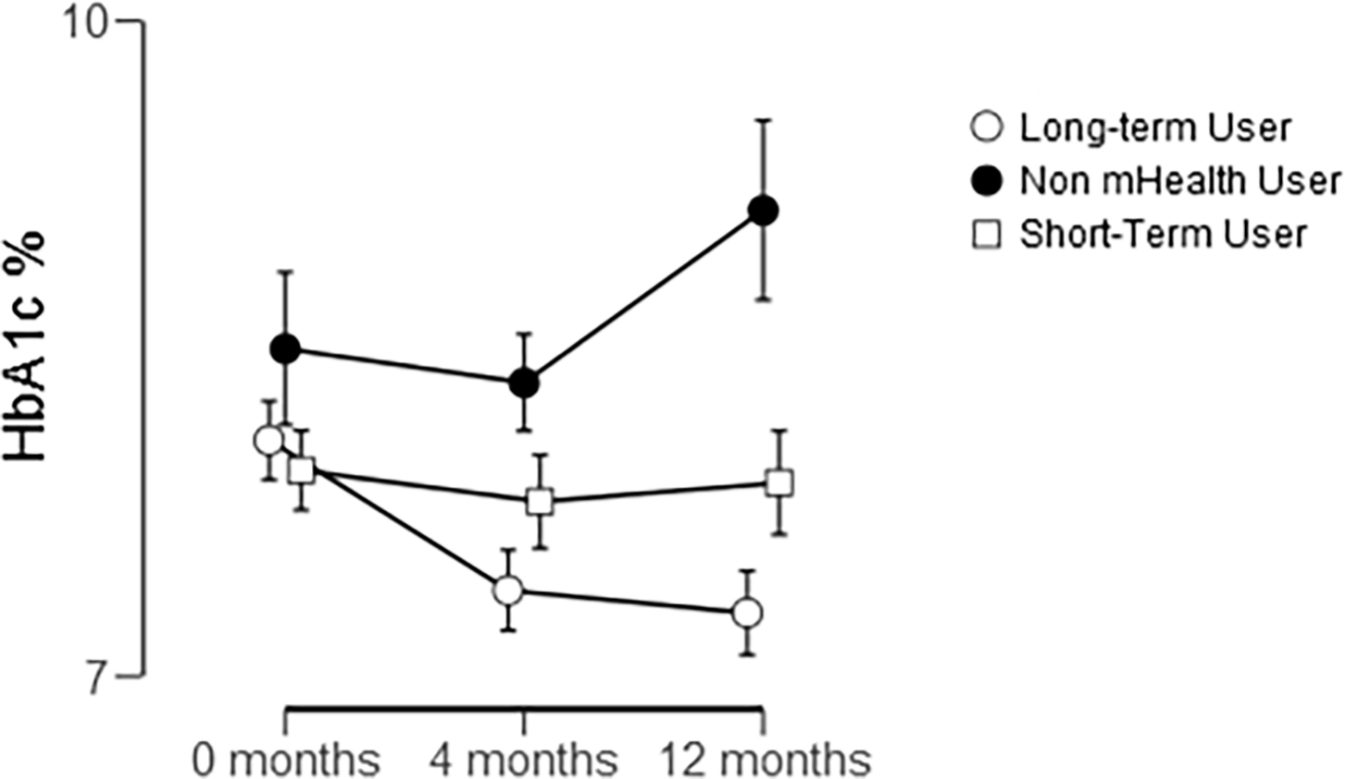
Comparison of HbA1c between users grouped by duration of FTA use. Error bars denote standard error of the mean.

According to a repeated-measure ANOVA among the 101 participants, time, i.e. the 12 months, did not affect HbA1c , F(2, 148) = .541, *P* = .583, η^2^ = .007. However, the Non-users, Short-term and Long-term users differed in change in HbA1c, F(2, 74) = 3.794, *P* = .027, η^2^ = .093. Among the Non-mHealth users and Short-term users were drop-outs, reducing the N in this analysis to n = 9 for non mHealth users, n = 7 for Short-term users, and n = 61 for Long-term users. The data does not change if one uses only the 0 and 4 months where there are slightly fewer dropouts. To compare specifically which groups differed from one another, a pair-wise comparison, or post-hoc test, was run. This showed that the only difference between groups was between the Non-mHealth users, who increased their HbA1c by 0.33%, and the Long-term users, who reduced their HbA1c by -0.86%, *P* = .021, Cohen’s d = .311. Short-term users did not differ from either of the other two groups. The interaction between time and group did not significantly impact change in HbA1c, F(4,148) = 1.26. *P* = .288, η^2^ = .033 (Fig 3). Note that the participants, in general, did not achieve the target (Norwegian) of achieving an HbA1c below 7mmol/L during the mHealth intervention.

### Exploration of Long-term users’ mHealth data and logs

We explored and compared the usage logs and data of the 61 participants who engaged in the intervention for three or more continuous months.

Baseline self-reported SMBG did correlate positively with the number of BG registrations (Pearson’s r = .579, *P* < .001, CI [.383; .725]), BG navigations (Pearson’s r = .436, *P* < .001 CI [.207; .62]), D/E registrations (r = .339, *P* = .008, CI [.095; .544]), and Goals (r = .409, *P* = .001, CI [.176; .599]), but not with D/E navigations (r = .229, *P* = .076, CI [-.024; .455]), made during the study. In contrast, change in HbA1c did not correlate with number of interactions spent on mHealth device functionalities, (all *P* > .15).

**Fig 4:**
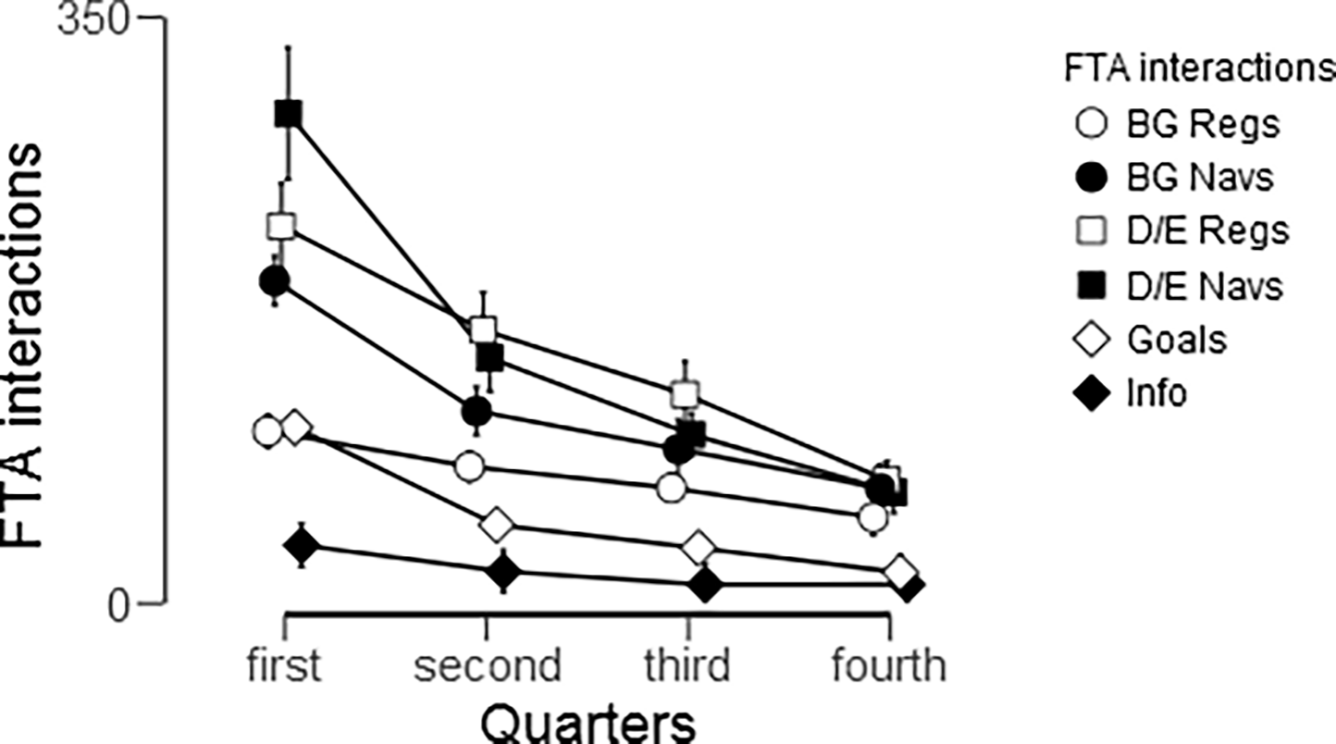
Used functionalities of the FTA per quarter. Error bars denote standard error of the mean (SEM).

Use of the FTA differed significantly both within and between individual functionalities over time. Fig 4 illustrates the results of a repeated measures ANOVA (Greenhouse-Geisser correction for sphericity violation), which revealed that total use significantly decreased over the four quarters: F(1.642, 98.528) = 45.02, *P* < .001, η^2^ = .429. Participants used the FTA mostly for diet / exercise (D/E) registration and navigations. Post-hoc tests revealed that the steepest decline is from first to second quarter with a decrease in 64.91 interactions on average (t = 9.234, *P*< .001), and for navigations in general. Use overall plateaued after this first quarter, with differences of fewer than 25 interactions between successive quarters (all *P* < .001). However, BG registrations were more consistent over the 1-year intervention, with the greatest difference was 21.51 BG registrations (t = 3.202, *P*< .05, effect size = .410) between the first and second quarter, whereas for example, participants decreased their use of D/E registrations significantly more—by 63.44 (t = 3.344, *P* < .01, effect size = .428)—over the same time. The use of each of the six functionalities differed significantly from one another in total (F(1.62, 97.2), *P* < .001, η^2^ = .202), as well as from one quarter to another: F(3.213, 192.795) = 13.26, *P* < .001, η^2^ = .181.The only functionalities that did not significantly differ were D/E Regs and D/E Navs, and BG Navs compared to D/E Regs and D/E Navs.

**Fig 5:**
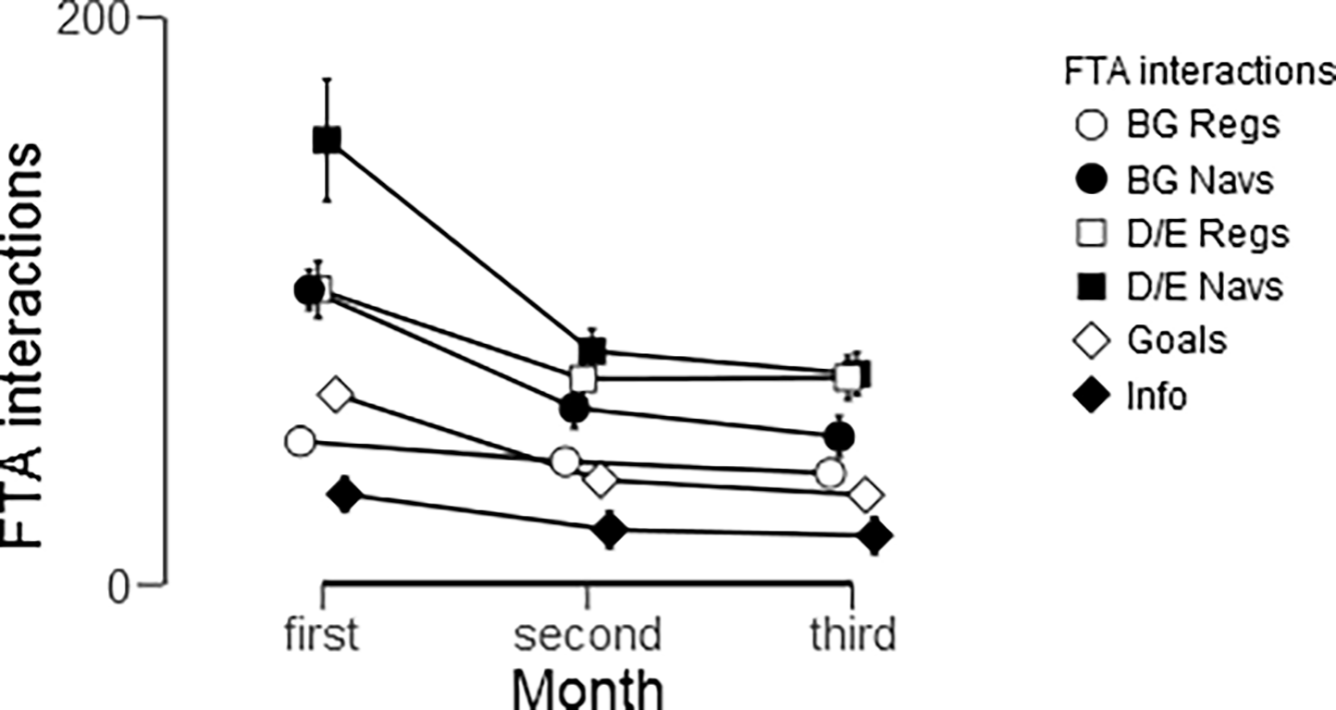
FTA usage over the first three months among the 61 Long-term users. Error bars denote standard error of the mean (SEM).

Next, we looked more closely at the first 3 months to identify where the reduction in FTA usage occurred the most (Fig 5). Overall, the same trends were found in use of the FTA between months as between quarters. In other words, use of the FTA differed significantly between these first three months (F(1.386, 83.14) = 23.545. *P* < .001, η^2^ = .282). Participants used the functionalities the most in the first month (461.2 ± 63) with a significant drop by 212.25 interactions (t = 5.022, *P* < .001, effect size = .643) during the second month. This was especially true for D/E navigations, which dropped by 35.374 interactions, on average, after the first month (t = 8.158, *P* < .001, d = 1.044). The plateau in use of the FTA actually began after the second month, as the second month did not differ from the third (t (60) = 1.379, *P* = .519, d = .177). However, BG registrations and Disease Information navigations were stable throughout the first 3 months with less than a 15 interaction difference between successive months (P < .05, except Disease Information Navigations between the second and third months). In addition, all functionalities differed from each other (all *P* < .01), with the exception of BG Regs and Goals which did not differ significantly.

We explored the barriers and opportunities for analysis of patients’ self-measured blood glucose values by comparing the in- and out-of-range values to number of registrations taken using the LifeScan blood glucose meter. Figure A in S4 Text demonstrates the great variability in frequency of SMBG both within and between participants over quarters of the study. S1 Table displays the significant Pearson Correlations between in-range BG measurements and goals functionality interactions for most quarters as well as in-range BG measurements and HbA1c for three of the four quarters.

### Identifying clusters and patterns from mHealth usage

The FTA offered six main functionalities and participants differed in their usage. Among the 61 Long-term users we performed a cluster analysis (k-means with at least N = 5 in a cluster) of the six functionalities: BG registrations, BG navigations, D/E registrations, D/E navigations, goals, and disease information. Cluster analysis yielded two clusters differing in their usage patterns (Fig 5), one dominated by diet/exercise registrations and navigations (n = 16), while the other cluster was dominated by BG registrations and navigations, as well as overall navigations (n = 40). Five participants were grouped in two very small clusters and were not included in further analysis.

**Fig 6:**
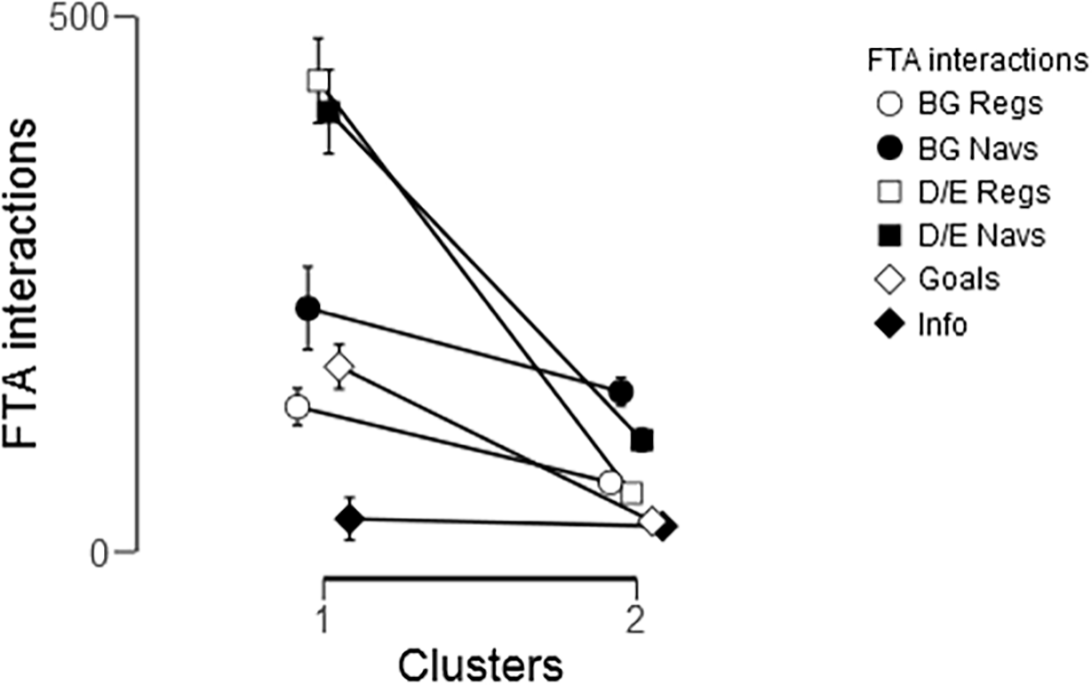
Comparison of the six interaction types between Cluster 1 (diet/exercise functionalities) and Cluster 2 (blood glucose functionalities and overall navigations) for the whole study period.

Repeated measure ANOVA confirmed that there was a significant difference between the use of the individual functionalities: F(2.987, 161.325) = 59.79, *P* < .001, η^2^ = .392, as well as use of each functionality over time between the clusters (F(2.987, 161.325) = 38.88, *P* < .001, η^2^ = .25). As can be observed in Fig 6, the only interactions in which these two clusters did not differ were for BG navigations, in total (*P* = .302) and between quarters (*P* = .129), and for Disease Information navigations, in total (*P* = .398) and between quarters (*P* = .689).

**Fig 7:**
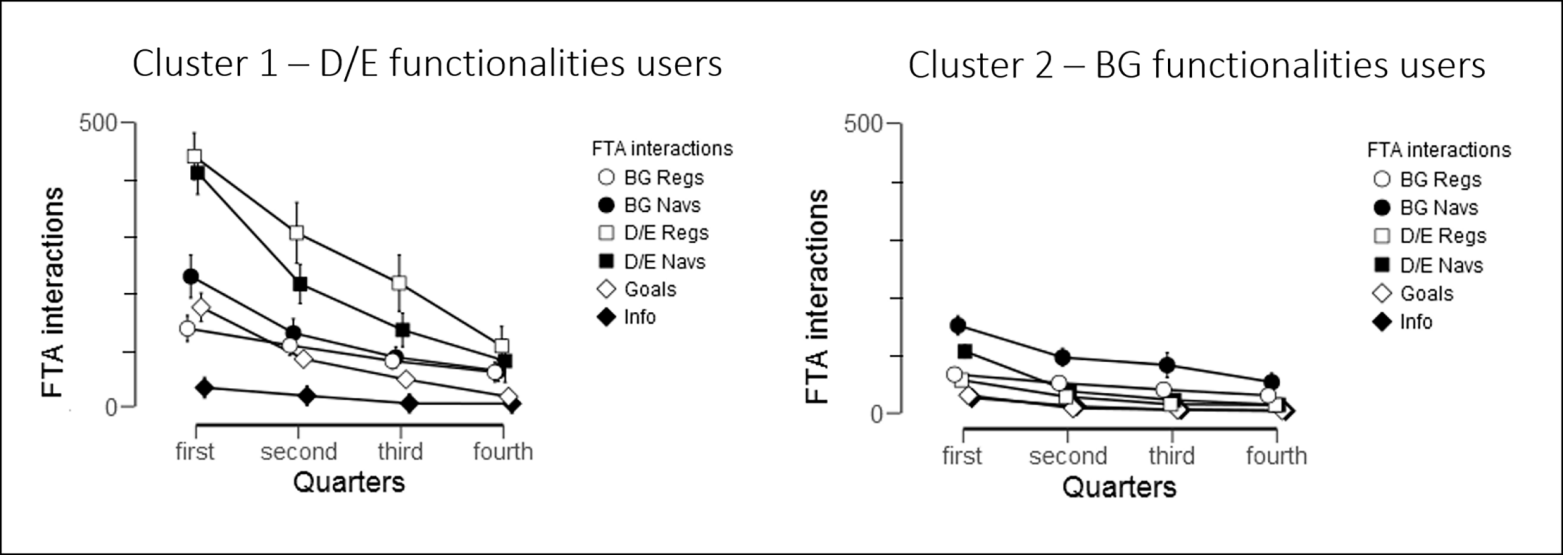
Distributions of functionalities used between Cluster 1 and Cluster 2 over the four quarters of the year. Error bars denote standard error of the mean (SEM).

Not only did Cluster 1 use the mHealth intervention more than Cluster 2 throughout the study, participants in this group also spent more time on interactions with goals functionalities in total (F(1,53) = 54.54, *P* < .001, η^2^ = .507) and between quarters (F(1.815, 96.211) = 42.47, *P* < .001, η^2^ = .241) (Fig 7). During the study, participants in Cluster 2 drastically decreased their use of goals, disease information and registration of diet/exercise. However, Cluster 1 was more consistent in their use of all functionalities overall. This can be seen in S5 Table, which details these changes in use over time by comparing percentages of functionalities used per quarter between and within each cluster. Of note is that while Cluster 1 spends most interactions on D/E activities, they still maintain their use of BG registrations, ranging from between 10%-16% per quarter, and BG navigations, ranging from between 12%-16% per quarter. On the other hand, while not statistically significant, Cluster 2 seems to increase BG activities while decreasing all other activities over the quarters.

**Fig 8:**
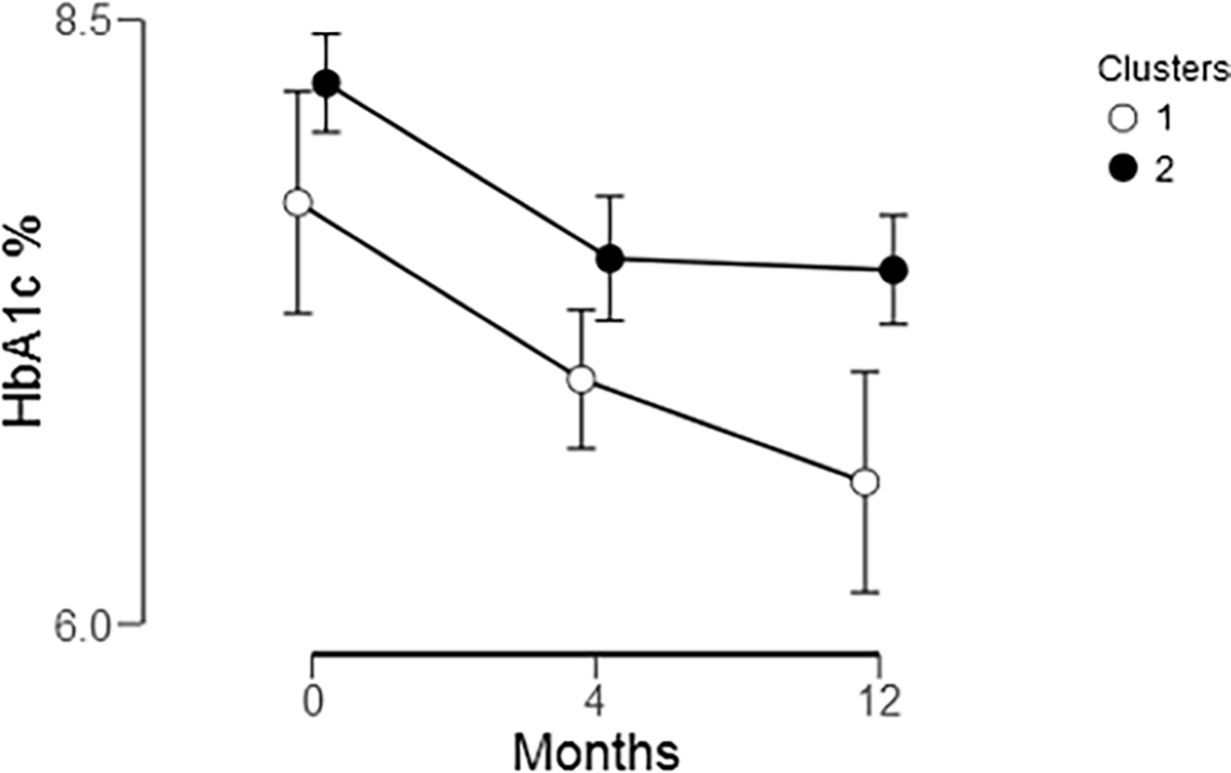
Comparison of change in HbA1c between Cluster 1 (D/E users, empty circles) and Cluster 2 (BG-users, filled circles) over baseline, 4- and 12-months.

Comparing the two groups, we found a non-significant group difference in HbA1c over the duration of the intervention (F(1, 55) = 3.642, *P* = .062, η^2^ = .062) (Fig 8). There was a significant difference between months (F(2, 110) = 5.043, *P* = .008, η^2^ = .084) but not between groups over time (F(2, 110) = .298, *P* = .743, η^2^ = .005). While not statistically significant, the group using the D/E functionalities of the FTA improved HbA1c over the course of the study, whereas the group using mainly the BG functionalities showed improvement during the first half of the year, but did not further improve in the second half of the year.

### Adverse events

While there were no clinical adverse events reported during the intervention, participants did report some technological issues. Of impact to this analysis were issues with the Bluetooth transfer of BG registered measurements from the BG meter to the Diabetes Diary app. This may have caused frustration and additional psychological stress and time when participants tried to engage in self-management during the intervention. While BG registrations were among the more stable types of interactions, it was postulated by Holmen et. al. that this may have discouraged participants from using that functionality or the FTA as a whole [26]. However, all participants were informed of these possibilities before the study began, including the possibility to get support via phone.

## Discussion

Theory-based evaluation is typically used to evaluate program interventions, such as education or, business management programs [12]. In fact, theory-based diabetes program interventions are common, but are characterized by structured instruction, external motivation and collaboration with clinical staff or support groups [28, 29]. However, programs and individuals’ diabetes self-management are similar in that they A) involve complex interactions and B) require that intervention evaluators acknowledge and understand these complex interactions. Therefore, we argue that it is appropriate to consider this presented approach as a supplement to hard clinical measures for mHealth studies. However, to the best of our knowledge, this study is the first to approach analysis of mHealth usage-logs by grouping such data based upon behavioural theories.

The insights from using the presented approach on this study are two-fold. The first is related to patients’ change in use of the mHealth intervention and the effect on their health. The second is related to what was learned about the proposed methods and approaches for analysing the impacts of the mHealth devices. As described below, additional data is required to properly interpret the impact of mHealth in self-management intervention studies. Therefore, the presented analysis should only be considered as a preliminary study of log data.

### Main results

90% of an iceberg’s mass is not noticed at first sight–the same can be said for mHealth interventions. First analysis of the data, based upon intervention groups, revealed no significant difference in change in HbA1c [30]. However, this additional analysis of actual and detailed mHealth logs revealed that those who did not use the mHealth tools increased their HbA1c over the course of the study while those who did use these tools significantly decreased their HbA1c. For all FTA users, the observed decrease in HbA1c between baseline and 4 months and the increase between 4- and 12-months suggests that the impact of using the FTA for 3+ months actually occurs during the first 4 months. This is just a coarse preview of the kind of relevant information that we can gain by categorizing users and reassessing outcomes based on actual usage.

Analysis of usage-patterns, for all mHealth users, and the comparison of groups based on functionalities used, revealed much more about how patients chose to engage in the intervention and their health over time than originally thought possible. By splitting up participants into similar groups based upon their own preferred use of the intervention, we were able to better understand the opportunities and limitations of how mHealth logs can reflect realistic and varied self-management habits. Comparison of how the functionalities were used by each cluster confirmed that individuals did in fact use the functionalities differently, which should be accounted or adjusted for in future analysis of mHealth interventions. Over the year, most patients fell into the blood glucose management cluster, and only 16 out of the 61 (or, when considering all of the users involved in the study, 101) patients also used the FTA’s lifestyle functions, i.e. diet and exercise registrations and navigations, significantly. Similarities also provide insights as to common approaches to treatment that may aid in adherence to clinical recommendations. For example, both clusters were similar in their use of goals functionalities in total–the main difference being when they used these functionalities (S2 Table). This information can be used to design improved mHealth interventions that encourage experiential learning by reinforcing the functionalities that patients already use and encouraging the use of un-used functionalities.

Further consideration of temporal relationships between usage of each functionality revealed that the number of interactions, and not time spent, with mHealth tools seem to be more suggestive of sustained mHealth use, as was demonstrated by the analysis of mHealth use related to goals (S3 Text). Those who did use the devices, their use significantly decreased after the first months suggesting that the first month reflects the novelty-effect of a new device. Therefore, the following months may have been more reflective of the realistic day-to-day use of mHealth devices for self-management. However, the assumed more familiar functionalities, such as BG registrations, were more consistent throughout the course of the study.

These logs not only provided insights about patients’ self-management habits, but could potentially provide a better understanding of how their health changes through their own-recorded health measures in self-management. For example, self-recorded BG values are informative of a patient’s health. However, inconsistency and lack of sufficient data limited our ability to suggest conclusive outcomes related to mHealth use and participants’ health (S4 Text). Further, we demonstrate how this approach to analysing usage logs can complement traditional measures. For example, the use of the goals functionalities suggested not only a relationship on both HbA1c and number of In-Range BG values over time, but also future and more sustained use of the mHealth functionalities (Table A in S3 Text).

### Experiences and future directions

We don’t know what we don’t know. Unless we ask the right questions in research, we cannot hope to achieve understanding of any endeavour regarding health interventions. In order to successfully measure the impacts of complex interventions such as mHealth interventions, we must look more deeply into how patients use—and differ in their use—of self-management technologies.

Therefore, we endeavoured to explore new concepts related to usage log analysis in this paper. In doing so, we aimed to provide accounts of practical and useful lessons learned and recommendations for future mHealth interventions. In Table 3, we summarize the main implications of the presented analysis for both research efforts and clinical practice.

**Table 3:** Aims, lessons learned and recommendations regarding analysis of usage-logs generated from the presented analysis.

Set	Aim	Lessons learned	Recommendations
1	To suggest and test a way of grouping log-data based on theories of human behaviour, to improve upon the tradition of summative analysis.	By grouping usage logs into “registrations” and “navigations” we were able to more easily and meaningfully identify how patients change their interactions with the mHealth devices.	When combined with traditional measures, established theories from complementary science fields, e.g. psychology, should be used to provide additional insight for mHealth intervention studies.
2	To explore what log-data can tell us about patients’ experience or relationship with the intervention technologies.	• The reduction in usage after the first month demonstrated the “novelty effect” of this technology. • Sustainable use, past the novelty effect, are dependent on relevant and easy-to-use functions.	• Analysis should consider and account for the “novelty effect” as a “run in” period, during which patients become more familiar with a technology before the intervention begins. • Automated functionalities, e.g. automatic registration of physical activity via Bluetooth from a wearable sensor, should be incorporated into the intervention when possible.
3	To suggest how researchers can tailor administration of the intervention to patients’ preferred use of the mHealth technologies.	The cluster analysis demonstrated that individuals indeed use mHealth tools differently based on the focus, or own priorities, of their self-management.	Reminders or recommendations for continued use and self-management practice can be tailored based on usage patterns of each patient during the first 3-months.
4	To propose a solution to achieve adequate data-collection.	The variability both within and between participants’ use was expected, and can be seen as a realistic representation of self-management amongst those with Type 2 diabetes.	Suggest minimum mHealth usage requirements for intervention studies to make data collection more consistent and reliable.
5	To determine how research and analysis can approach patient collected health measures.	Self-collected health data, such as BG values, diet and exercise, can supplement health measures collected at the point-of-care by providing details of health change between consultations. However, consistency and reliability of the data is required.	While lifestyle measures such as diet and exercise can be episodic and without schedule, measures such as SMBG should be done on a consistent schedule to ensure their comparability over time and two other measures during interventions.
6	To determine what more is needed to understand not only what and how, but also why patients choose to self-manage.	Usage logs are a valuable resource for understanding how use of diabetes mHealth tools change during the intervention. However, why changes occurred during the intervention period were not clear.	Related and complementary questionnaires include, e.g. Patient Activation Measure [31], Health Education Impact Questionnaire [32], Patient Health Locus of Control [33], which measure motivation and patients’ intention to engage in their health, as well as the Health Care Climate Questionnaire [34], which may provide insights as to the impact of the therapeutic relationship related to not only engagement in self-care and health outcomes but also mHealth use.

### Strengths and limitations

This presented analysis of the RENEWING HEALTH study is underpowered. However, barriers, limitations and setbacks are only as negative as your reaction to them. In fact, limitations experienced during this study provided greater insight for how to not only improve future mHealth interventions but also how to approach their evaluation. Of those who received the mHealth intervention, 29 participants (30%) did not use it once after the start-up meeting, which rose to 45 participants not using it after the first three months. Furthermore, all participants reduced their usage of the mHealth tools significantly over time. This led us to question where the barriers for sustainable use occurred and how we could address these in the next iteration of mHealth intervention studies. Analysis of the usage-logs, such as those actions related to the manual entry of diet/exercise data, revealed that time-consuming or burdensome usage-requirements discouraged many from long-term engagement with those functionalities. However, those in Cluster 1, who used the FTA largely for diet/exercise management, also reduced their HbA1c. While this is both encouraging and telling of how usage can be associated with health change, we cannot rely solely on statistical testing of small and diverse samples to conclude on the impact of any mHealth intervention. Another point that is both positive and negative for future studies is that this technology it still changing rapidly. In fact, the mHealth devices used in this study had become almost outdated by the end of the trial, which may have contributed to frustration and a steep decline in use over the year.

These trends raise two common plights of research interventions: 1) the desire or habit to trust statistical output at face value, without scepticism and assessment of the reliability of the data itself compared to real-world scenarios and 2) sustaining use of the intervention so as to collect enough consistent and reliable data to produce conclusive results. In order to both improve participants’ experience during interventions and also ease the burdens of self-management instead of creating them, the use of mHealth technologies should be less time-consuming, more relevant and provide greater reinforcement of beneficial habits than standard modalities. In relation, participants were not given long-term reinforcement for when and how to use the devices. The benefit of providing such reinforcement could be two-fold. First, participants may feel more supported and engaged. Second, we as researchers may be able to reduce variability and inconsistency in device usage, which would facilitate actionable statistical analysis and more insightful interpretation of the intervention results.

## Conclusion

Today’s mHealth technology can allow researchers and health-care practitioners to not only better understand but also better reinforce patient’s self-management behaviours—but we need to adapt research practices to keep up. Analysis of quarterly accounts of usage-logs, differentiated by functionality and purpose, illustrated that clinical research can benefit from studying usage patterns in such a way that provide meaningful and actionable information beyond the typical conclusion of “further studies are needed”. This is evident in the comparison between previously reported results of the Renewing Health project versus the presented study of log data. The previous study used total measures of logs based on originally assigned intervention groups and demonstrated no difference in use of the app or HbA1c’s between intervention groups. However, in the present study using log data, by analysing the impact of the app based on how individuals used the apps functionalities, we were able to identify for which users the mHealth intervention yielded a significant improvement in HbA1c. We propose the presented exploratory analysis as a novel supplement to the traditional hard-measures of diabetes health and self-management, and encourage others to use, comment, suggest and discuss this approach. We also aim to apply this approach both retroactively to the now completed Tailoring Type 2 Diabetes Self-Management Study [35] data-set and proactively to the design and testing of the mHealth data-sharing intervention in the Full Flow of Data between Patients and Health Care Systems Project’s [36].

## Supporting information

S1 File RENEWING HEALTH study protocol. (PDF)

S2 File CONSORT checklist. (DOC)

S3 File Analysed data. (XLSX)

S1 Text How-to: selection of relevant data from usage logs. (DOCX)

S2 Text K-means clustering. (DOCX)

S3 Text Minutes vs. interactions with goals functionalities. (DOCX)

S4 Text Patients’ self-registered blood glucose values. (DOCX)

S1 Table Results of Pearson Correlations run between interactions with the Goals functionalities within the app and In-Range BG measurements per quarter of the year (n = 61). (DOCX)

S2 Table Percentage distribution of the six FTA interaction types used by clusters 1 (n = 16) and 2 (n = 40) for each quarter. (DOCX)
