# Supplementary material for: Analysing mHealth usage logs in RCTs: Explaining participants’ interactions with type 2 diabetes self-management tools
Source: PLoS One. 2018 Aug 30;13(8):e0203202. doi: 10.1371/journal.pone.0203202 (PMC6117049; doi:10.1371/journal.pone.0203202)
Supplement: S1 Text — (DOCX) [file pone.0203202.s004.docx]

**S1 Text. How-to: selection of relevant data from usage logs**

Log data was extracted from CSV files exported from the app, into Microsoft Excel where logs were included based upon their relevance to the presented analysis approach. As such, logs related to back-end administrative functionalities, automatic app-closing and updates etc. were excluded. Only interactions that directly related to the self-management functionalities of the system were included, i.e. functionalities related to blood glucose, diet, exercise, goals and disease information material.

Minutes spent on each screen of the mHealth app were calculated based upon the time stamps for each logged event. However, it is important to note that because BG registrations were made on a separate device connected to the mHealth app, yet stored together with the logs of other mHealth interactions made on the app, calculations for minutes spent with the mHealth app will exclude those related to registering blood glucose data. Therefore, only minutes spent entering the other data types, reviewing data, interacting with goal-related screens and interacting with the home screen are included.
