# Supplementary material for: Analysing mHealth usage logs in RCTs: Explaining participants’ interactions with type 2 diabetes self-management tools
Source: PLoS One. 2018 Aug 30;13(8):e0203202. doi: 10.1371/journal.pone.0203202 (PMC6117049; doi:10.1371/journal.pone.0203202)
Supplement: S2 Text — (DOCX) [file pone.0203202.s005.docx]

**S2 Text. K-means Clustering**

The purpose of using the k-means clustering algorithm is to identify naturally occurring subgroups within a dataset based upon the closeness of data points to central point. The number of central means (k) is given to the algorithm by the analyst, based upon their estimated number of clusters. The distance of each data point determines which cluster or subgroup that data point belongs to. However, the algorithm’s identification of each cluster, and their central mean, was automatically adjusted until the mean distances between each data point and the central point four minimized. In other words, the algorithm provides the best fit for each data point into each cluster so that the similar data points end up in the same cluster.

In the presented case, these data points are participants and the central means are calculated based upon participants’ total app usage in the first quarter. We had six possible clusters (Table 1), we explored starting with k=6. Results with clusters where too few participants were not retained. That is, we aimed for cluster sizes between 10 to 40 participants. With k=4 two main clusters and 2 smaller clusters with n<5 participants were identified. For the two large clusters, n=16 and n=40, the usage logs of those patients in each cluster were compared.
