# Supplementary material for: Analysing mHealth usage logs in RCTs: Explaining participants’ interactions with type 2 diabetes self-management tools
Source: PLoS One. 2018 Aug 30;13(8):e0203202. doi: 10.1371/journal.pone.0203202 (PMC6117049; doi:10.1371/journal.pone.0203202)
Supplement: S3 Text — (DOCX) [file pone.0203202.s006.docx]

**S3 Text. Minutes vs. interactions with Goals functionalities**

Pearson Correlations delved deeper into the combination between and order in which mHealth functionalities were used over time. The number of interactions with the Goals functionalities significantly correlated positively with the other mHealth functionalities consistently throughout the study. However, time spent on the Goals screens did not correlate significantly with use of the BG Registrations and Reviews nor with Disease Information Reviews (Table A). These results were consistent when logs were split into quarters. This suggests that action and not time spent with the mHealth devices is more telling about sustained use of mHealth tools and their different functionalities. However, neither interactions nor time spent on functionalities were significantly correlated with change in HbA1c (as a continuous variable), nor HbA1c at baseline 4- or 12-months.

**Table A.** Pearson’s Correlations between each of the six functionalities and minutes spent on goals functionalities (n=61) over the year.

|  |  | Total Goals (mins) | Total Goals | Total BG Regs | Total BG Navs | Total DE Regs | Total DE Navs | Total Info Navs |
| --- | --- | --- | --- | --- | --- | --- | --- | --- |
| Total Goals (mins) | *Pearson's r* | *—* |  |  |  |  |  |  |
|  | *p-value* | *—* |  |  |  |  |  |  |
| Total Goals | *Pearson's r* | *-0.089* | *—* |  |  |  |  |  |
|  | *p-value* | *0.496* | *—* |  |  |  |  |  |
| Total BG Regs | *Pearson's r* | *0.082* | *0.634* | *—* |  |  |  |  |
|  | *p-value* | *0.531* | *< .001* | *—* |  |  |  |  |
| Total BG Navs | *Pearson's r* | *0.1* | *0.472* | *0.577* | *—* |  |  |  |
|  | *p-value* | *0.441* | *< .001* | *< .001* | *—* |  |  |  |
| Total DE Regs | *Pearson's r* | *-0.117* | *0.889* | *0.637* | *0.374* | *—* |  |  |
|  | *p-value* | *0.369* | *< .001* | *< .001* | *0.003* | *—* |  |  |
|  |  |  |  |  |  |  |  |  |
| Total DE Navs | *Pearson's r* | *-0.099* | *0.737* | *0.621* | *0.32* | *0.899* | *—* |  |
|  | *p-value* | *0.447* | *< .001* | *< .001* | *0.012* | *< .001* | *—* |  |
| Total Info  Navs | *Pearson's r* | *0.391* | *0.295* | *0.412* | *0.474* | *0.218* | *0.26* | *—* |
|  | *p-value* | *0.002* | *0.021* | *< .001* | *< .001* | *0.092* | *0.043* | *—* |

*Note*: * *p*<0.05, ***p*<0.01, *** *p*<0.001
