# Supplementary material for: Analysing mHealth usage logs in RCTs: Explaining participants’ interactions with type 2 diabetes self-management tools
Source: PLoS One. 2018 Aug 30;13(8):e0203202. doi: 10.1371/journal.pone.0203202 (PMC6117049; doi:10.1371/journal.pone.0203202)
Supplement: S4 Text — (DOCX) [file pone.0203202.s007.docx]

**S4 Text. Patients’ self-registered blood glucose values**

Here we describe the process of analysing the self-registered blood glucose measures by determining if the values were in- or out- of the recommended range (i.e. 4-10 mmol/L), expressed as proportion of all BG registrations they made. This provided us an additional opportunity to understand the intervention’s impact on participants’ health. While HbA1c values were measured during consultations with clinicians at certain intervals during the study, self-management of BG registrations (SMBG) can be used as a more frequent measure of diabetes health if the measures are consistent and reliable. However, despite the potential benefit of such data, participants’ number of BG registrations declined over the year and varied considerably as seen on the large error bars in Figure A. Only N=39 participants provided data for all four quarters. Furthermore, the average number of BG measurements in the fourth quarter (65.63) is approximately 66% of the total BG measurements taken in the first quarter (98.9), demonstrating too great a difference for comparability between quarters. Their In-Range proportion did not change over time, *F*(3,114) = .598, *P* = .618, η^2^ = .015. While statistical testing may not be the most appropriate, this trend does point to a relationship worth exploring, i.e. possibly by suggesting an agreed upon frequency of SMBG, e.g. a minimum of once before breakfast for a certain number of days each week, for participants in future studies.


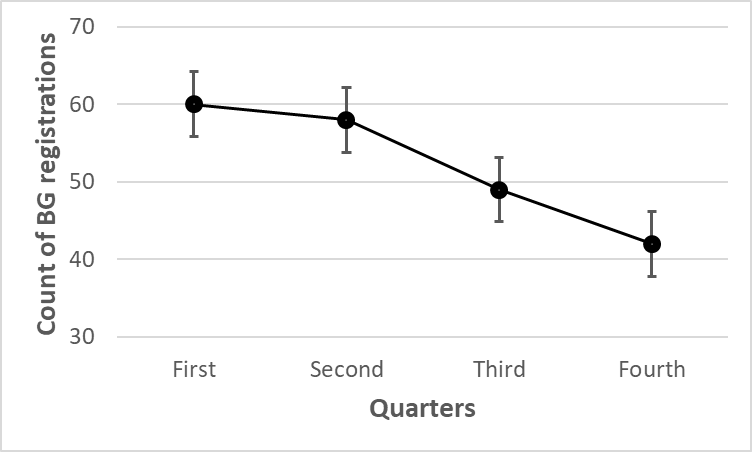


**Figure A.** Number of BG registrations for long-term users (n=61) per quarter of the year.
