# Supplementary material for: Analysing mHealth usage logs in RCTs: Explaining participants’ interactions with type 2 diabetes self-management tools
Source: PLoS One. 2018 Aug 30;13(8):e0203202. doi: 10.1371/journal.pone.0203202 (PMC6117049; doi:10.1371/journal.pone.0203202)
Supplement: S1 Table — (DOCX) [file pone.0203202.s008.docx]

**S1 Table.** Results of Pearson Correlations run between interactions with the Goals functionalities within the app and In-Range BG measurements per quarter of the year (n=61).

|  |  | Q1 In-Range | Q2 In-Range | Q3 In-Range | Q4 In-Range | Q1 Goals | Q2 Goals | Q3 Goals | Q4 Goals | HbA1c at 0 | Change in HbA1c | Age | Duration |
| --- | --- | --- | --- | --- | --- | --- | --- | --- | --- | --- | --- | --- | --- |
| Q2 In-Range | *Pearson's r* | *0.815* | *—* |  |  |  |  |  |  |  |  |  |  |
|  | *p-value* | *< .001* | *—* |  |  |  |  |  |  |  |  |  |  |
| Q3 In-Range | *Pearson's r* | *0.772* | *0.854* | *—* |  |  |  |  |  |  |  |  |  |
|  | *p-value* | *< .001* | *< .001* | *—* |  |  |  |  |  |  |  |  |  |
| Q4 In-Range | *Pearson's r* | *0.587* | *0.674* | *0.615* | *—* |  |  |  |  |  |  |  |  |
|  | *p-value* | *< .001* | *< .001* | *< .001* | *—* |  |  |  |  |  |  |  |  |
| Q1 Goals | *Pearson's r* | *0.687* | *0.631* | *0.642* | *0.394* | *—* |  |  |  |  |  |  |  |
|  | *p-value* | *< .001* | *< .001* | *< .001* | *0.012* | *—* |  |  |  |  |  |  |  |
| Q2 Goals | *Pearson's r* | *0.554* | *0.61* | *0.574* | *0.241* | *0.791* | *—* |  |  |  |  |  |  |
|  | *p-value* | *< .001* | *< .001* | *< .001* | *0.135* | *< .001* | *—* |  |  |  |  |  |  |
| Q3 Goals | *Pearson's r* | *0.553* | *0.597* | *0.61* | *0.034* | *0.663* | *0.886* | *—* |  |  |  |  |  |
|  | *p-value* | *< .001* | *< .001* | *< .001* | *0.836* | *< .001* | *< .001* | *—* |  |  |  |  |  |
| Q4 Goals | *Pearson's r* | *0.528* | *0.546* | *0.6* | *0.037* | *0.589* | *0.722* | *0.926* | *—* |  |  |  |  |
|  | *p-value* | *< .001* | *< .001* | *< .001* | *0.82* | *< .001* | *< .001* | *< .001* | *—* |  |  |  |  |
| HbA1c at 0 | *Pearson's r* | *-0.257* | *-0.301* | *-0.272* | *-0.37* | *-0.165* | *-0.086* | *-0.113* | *-0.105* | *—* |  |  |  |
|  | *p-value* | *0.049* | *0.022* | *0.068* | *0.019* | *0.204* | *0.511* | *0.385* | *0.419* | *—* |  |  |  |
| Change in HbA1c | *Pearson's r* | *0.041* | *0.091* | *0.007* | *0.087* | *0.051* | *-0.031* | *0.039* | *0.177* | *-0.373* | *—* |  |  |
|  | *p-value* | *0.756* | *0.498* | *0.965* | *0.591* | *0.699* | *0.81* | *0.767* | *0.173* | *0.003* | *—* |  |  |
| Age | *Pearson's r* | *0.1* | *0.331* | *0.27* | *0.377* | *0.1* | *0.05* | *0.07* | *0.076* | *-0.184* | *0.223* | *—* |  |
|  | *p-value* | *0.453* | *0.011* | *0.07* | *0.017* | *0.444* | *0.7* | *0.593* | *0.56* | *0.157* | *0.083* | *—* |  |
| Duration | *Pearson's r* | *0.026* | *0.035* | *0.262* | *0.472* | *0.128* | *0.066* | *-0.063* | *-0.051* | *0.166* | *-0.192* | *0.265* | *—* |
|  | *p-value* | *0.844* | *0.793* | *0.079* | *0.002* | *0.324* | *0.613* | *0.63* | *0.693* | *0.202* | *0.138* | *0.039* | *—* |
| SMBG | *Pearson's r* | *0.648* | *0.484* | *0.402* | *0.227* | *0.374* | *0.386* | *0.375* | *0.326* | *0.108* | *-0.144* | *0.093* | *0.17* |
|  | *p-value* | *< .001* | *< .001* | *0.006* | *0.159* | *0.003* | *0.002* | *0.003* | *0.01* | *0.406* | *0.269* | *0.474* | *0.189* |

*Note*: * *p*<.05, ***p*<.01, *** *p*<.001
