# Supplementary material for: Analysing mHealth usage logs in RCTs: Explaining participants’ interactions with type 2 diabetes self-management tools
Source: PLoS One. 2018 Aug 30;13(8):e0203202. doi: 10.1371/journal.pone.0203202 (PMC6117049; doi:10.1371/journal.pone.0203202)
Supplement: S2 Table — (DOCX) [file pone.0203202.s009.docx]

**S2 Table.** Percentage distribution of the six FTA interaction types used by clusters 1 (n=16) and 2 (n=40) for each quarter.

|  | Quarter | BG Regs (%) | BG Navs (%) | D/E Regs (%) | D/E Navs (%) | Goals (%) | Info (%) |
| --- | --- | --- | --- | --- | --- | --- | --- |
| Cluster 1 | 1 | 10% | 16% | 31% | 29% | 12% | 2% |
|  | 2 | 12% | 14% | 37% | 24% | 9% | 3% |
|  | 3 | 12% | 12% | 48% | 20% | 8% | 1% |
|  | 4 | 16% | 15% | 42% | 21% | 4% | 1% |
| Cluster 2 | 1 | 15% | 35% | 13% | 25% | 7% | 6% |
|  | 2 | 23% | 43% | 12% | 16% | 3% | 4% |
|  | 3 | 24% | 51% | 8% | 13% | 2% | 2% |
|  | 4 | 26% | 48% | 11% | 11% | 2% | 1% |
